# Investigation of the Utility of Features in a Clinical De-identification Model: A Demonstration Using EHR Pathology Reports for Advanced NSCLC Patients

**DOI:** 10.3389/fdgth.2022.728922

**Published:** 2022-02-16

**Authors:** Tanmoy Paul, Md Kamruz Zaman Rana, Preethi Aishwarya Tautam, Teja Venkat Pavan Kotapati, Yaswitha Jampani, Nitesh Singh, Humayera Islam, Vasanthi Mandhadi, Vishakha Sharma, Michael Barnes, Richard D. Hammer, Abu Saleh Mohammad Mosa

**Affiliations:** ^1^Department of Electrical Engineering and Computer Science, University of Missouri, Columbia, MO, United States; ^2^Center for Biomedical Informatics, University of Missouri, Columbia, MO, United States; ^3^Department of Health Management and Informatics, School of Medicine, University of Missouri, Columbia, MO, United States; ^4^Institute for Data Science and Informatics, University of Missouri, Columbia, MO, United States; ^5^Roche Diagnostics, F. Hoffmann-La Roche, Santa Clara, CA, United States; ^6^Department of Pathology and Anatomical Sciences, University of Missouri, Columbia, MO, United States

**Keywords:** clinical text de-identification, protected health information, NLP, named entity recognition, de-identification, conditional random field, data warehousing

## Abstract

**Background:**

Electronic health record (EHR) systems contain a large volume of texts, including visit notes, discharge summaries, and various reports. To protect the confidentiality of patients, these records often need to be fully de-identified before circulating for secondary use. Machine learning (ML) based named entity recognition (NER) model has emerged as a popular technique of automatic de-identification.

**Objective:**

The performance of a machine learning model highly depends on the selection of appropriate features. The objective of this study was to investigate the usability of multiple features in building a conditional random field (CRF) based clinical de-identification NER model.

**Methods:**

Using open-source natural language processing (NLP) toolkits, we annotated protected health information (PHI) in 1,500 pathology reports and built supervised NER models using multiple features and their combinations. We further investigated the dependency of a model's performance on the size of training data.

**Results:**

Among the 10 feature extractors explored in this study, n-gram, prefix–suffix, word embedding, and word shape performed the best. A model using combination of these four feature sets yielded precision, recall, and F1-score for each PHI as follows: NAME (0.80; 0.79; 0.80), LOCATION (0.85; 0.83; 0.84), DATE (0.86; 0.79; 0.82), HOSPITAL (0.96; 0.93; 0.95), ID (0.99; 0.82; 0.90), and INITIALS (0.97; 0.49; 0.65). We also found that the model's performance becomes saturated when the training data size is beyond 200.

**Conclusion:**

Manual de-identification of large-scale data is an impractical procedure since it is time-consuming and subject to human errors. Analysis of the NER model's performance in this study sheds light on a semi-automatic clinical de-identification pipeline for enterprise-wide data warehousing.

## Introduction

Clinical texts are vital components of electronic health records (EHR) and can be an enriched knowledge source for medical research. However, text-based medical records often contain potential patient identifiers and confidential information that must not be shared with third parties for ethical and legal reasons. The Health Insurance Portability and Accountability Act (HIPAA) in the United States requires removing patient-protected health information (PHI) from the medical records before sharing for secondary use (1). The PHI items include name, geographic location, phone number, social security number, medical record number, etc. (2, 3). The manual de-identification process of large-scale data is time-consuming, expensive, prone to error, and impractical. Therefore, a reliable automated de-identification system of clinical documents can be exceedingly valuable for healthcare research.

The application of natural language processing (NLP) on electronic health records (EHR) has a significant impact on biomedical and healthcare-related research. Named entity recognition (NER) is a fundamental task of clinical NLP. NER is the process of identifying entities of interest in a text. Although clinical NLP has been an area of increasing interest among researchers in recent years, NER is still a challenging task. A medical record consists of both coded data and unstructured texts. While the de-identification of coded data is straightforward, the de-identification of unstructured texts is posed with many challenges. For instance, lack of clinical narrative corpora because of privacy concerns is one major predicament in de-identifying clinical documents.

Most of the initial NER systems deployed rule-based approaches (4, 5). For classification and identification of named entities (NE), these systems utilized information lists as well as rules-based on syntactic-lexical patterns (6, 7). These approaches are considered to be highly efficient since they exploit language-related knowledge (8). On the other hand, these methods are expensive, domain-specific, and require human expertise in the domain. Hence, the rule-based system built for one domain cannot be transferred to another domain. These limitations have shifted the interests of researchers toward machine learning-based approaches.

There have been extensive efforts by researchers to explore machine learning algorithms in clinical NER. Many researchers have undertaken various strategies taking advantage of the existing infrastructure of machine learning algorithms to improve system performance. Existing literature shows the use of an ensemble of multiple machine learning methods (9, 10), hybrid machine learning models with high confidence rules (11), unsupervised model using clustering algorithms (12–14) in clinical NER applications with supervised machine learning algorithms engendering the best-performing systems.

There are multiple machine learning algorithms available to build a clinical NER model, such as conditional random fields (CRF), maximum entropy (ME), structured support vector machines (SVM) (15–17). These models are based on predefined features representing the multidimensional aspect of a text dataset exploited by learning methods to generate a model. CRF has been used in multiple top-ranked supervised NER models. However, supervised NER is highly sensitive to the proper selection of features. This motivated us to investigate how the performances of the de-identification models vary when built using different set of features.

In this study, we used pathology reports to build and test our de-identification model. Compared to radiology reports, clinical notes, and discharge summaries, information in pathology reports retains its value over a long time (18). The diagnoses in pathology reports form the base of many vital clinical research studies. For our de-identification model, non-small cell lung cancer (NSCLC) pathology reports were used. Lung cancer accounts for 27% of all the cancer deaths in the United States (19). NSCLC is the most lethal form of cancer globally as it causes more death than other forms of cancer combined (20). Hence, in cancer-related research, pathology reports of NSCLC patients have a unique position among other clinical documents. This study aims to determine the utility of features in building a CRF-based de-identification model using NSCLC pathology reports. The key contributions of this study are two-fold: (i) Firstly, our investigation will be helpful in choosing the proper set of features to build a de-identification NER model from a wide range of features at one's disposal, and (ii) secondly, we also propose a framework of a semi-automatic clinical de-identification pipeline which can play a significant role in an enterprise-wide data warehousing.

## Methods

### Dataset

We extracted 1,500 advanced (NSCLC) pathology reports of 421 patients from the University of Missouri Health Care EHR. All the patients were diagnosed with IIIB or a higher cancer stage with a diagnosis period between 2010 and 2018. The dataset consisted of four types of pathology reports, including cytology, surgical, thromboelastography, and peripheral blood smear. In this study, we identified six types of PHI from these unstructured text reports. The PHIs were NAME, DATE, HOSPITAL, LOCATION, PHONE, INITIALS, and ID number.

### NLP Toolkit

An NLP software, MITRE Identification Scrubber Toolkit (MIST), was used to annotate the PHIs in the pathology reports (21). These reports went through a two-step annotation process for tagging the protected health information as defined by HIPAA. At the first step, a data annotator manually annotated the reports. In the second step, a second annotator reviewed each report and made corrections if there was an error. Moreover, once the second annotator validated the first stage of the annotation, a python code was used to replace the annotated PHI items with relevant tags. For example, all the identified names and dates were replaced by [NAME] and [DATE], respectively. These de-identified text reports were reviewed again by a third reviewer to ensure that we have a completely annotated gold standard repository. If the third reviewer found any error, the second annotator was asked to fix it. Supervised NER models were built and tested by using this gold standard dataset.

Another NLP toolkit, Clinical Language Annotation, Modeling and Processing (CLAMP), was used to build the NER model (22). CLAMP enabled us to build a model by training Conditional Random Field (CRF) with multiple features both individually and as a combination of any number of those features.

The entire experiment was divided into two stages: Identifying the best feature set and determining the minimum size of the training set to achieve the best performance.

### Identifying Best Feature Set

**Figure 1:**
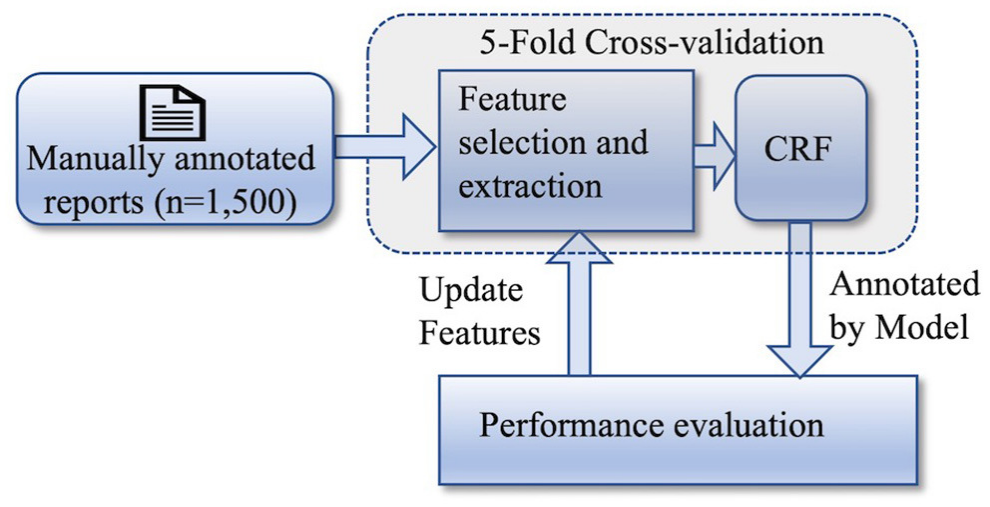
Workflow of the 1st stage of the experiment: identifying the best feature set.

In the first stage of our experiment, the objective was to determine which features make the NER model perform the best in annotating the aforementioned PHIs. Figure 1 shows the workflow of this stage of the experiment. A total of 10 features were explored in this study and a model was built using all of the manually annotated reports (*n* = 1,500) by extracting each of these features. The model was validated by applying the 5-fold cross-validation technique. The performances of all of the 10 models were evaluated by calculating the performance measures, such as precision (P), recall (R), and F1-score (F1). Based on the values of the performance measures, the best performing features were identified. Furthermore, we investigated the possibility of improving the model's performance by using a combination of features. Therefore, we built two more models, the first one by combining all the features and the second one by combining the best performing features. The following describes the features we explored in our study.

**Brown Clustering (BC)**, a class-based language model, is a hierarchical clustering algorithm that aims to maximize the mutual information of bigrams by clustering words. The significance of the hierarchical nature of clustering is that the word class can be chosen at several levels in the hierarchy. It enables the compensation for poor clusters with few words (23).

**N-gram (NG)** exploits the co-occurrence of other items before and after a named entity. Depending on the application, these items can be letters, syllables, or words. In a 2-gram model, the left and right neighbors of these annotated entities are checked, and the frequency of each pair of such neighbors is counted (24). The **prefix–suffix (PS)** feature is the prefix and suffix of words that can represent a specific type of NE.

**Random Indexing (RI)** is a dimensionality reduction method that compresses word-context co-occurrence matrices (25). In this method, each document or context is associated with an index vector, which is a sparse random vector with high dimensionality. Each word is also associated with a high dimensional vector of integers called a distributional vector. Initially, distributional vectors are set to zero, and whenever a particular word is encountered in a context, the distributional vector of that word is updated by adding the index vector of that context. Hence, words with similar distributional vectors are considered semantically related (25).

**Section (S)** feature is the section where a named entity exists. **Sentence pattern (SP)** uses CLAMP built-in rules and distinguishes the pattern of a sentence. **Word Embedding (WE)** is similar to BC and RI as it is a type of distributed word representation feature generated on unlabeled data. In **discrete word embedding (DWE)**, character level features are extracted at word level from a distributed representation, and it does not require knowledge of the syntactic or semantic structure of language (26). **Word Shape (WS)** identifies whether a word begins with an English letter, number, etc., or not. **Word regular expression (WRE)** is the regular expression patterns of words that may indicate a specific type of named entity.

### Determining the Minimum Size of Training Set

**Figure 2:**
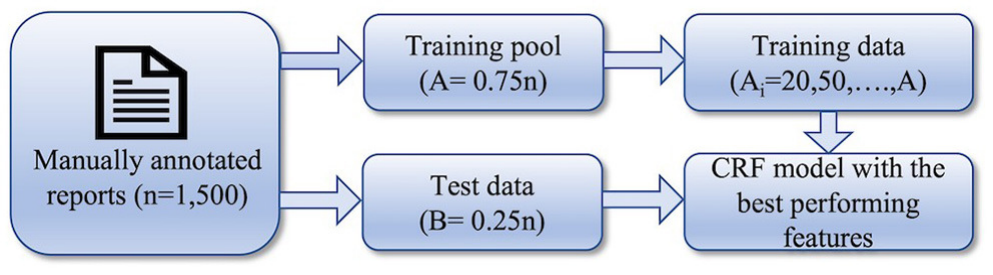
Workflow of the 2nd stage of the experiment: determining the minimum size of training set.

In the second stage, our goal was to observe the dependency of the model's performance on the variation of the number of training data and determine the minimum number of training data required to achieve the highest F1-score. The workflow of this stage of the experiment is shown in Figure 2. We split the entire dataset randomly into training pool and test data with 3:1 ratio. Multiple models were built using variable numbers of training data from the training pool. Using the best feature sets derived from the first stage, multiple models were built using a varying number of data points as part of the training process. On the first iteration, only 10 training data were used to build a model. On the subsequent iterations, the number of training data in the training set was increased (20, 50, 100, 200, 300, 400, 500, 600, 700, 800, 900, and 1,000). The same set of test data was used to evaluate the performance of all the models.

## Results

The performance measures to identify the best feature set are presented in Tables 1, 2. Table 1 shows precision, recall, and F1-score achieved for each PHI item by each feature. As expected, not all the features were equally effective in recognizing the named entities. NG outperformed all other features in annotating most PHI items by achieving the highest performance measures, as shown in Table 1. For instance, the model trained by NG yielded the highest performance measures (precision = 0.80, recall = 0.78, and F1-score = 0.79) in identifying NAME. PS and WS achieved the second-highest precision value (0.79). The second highest recall and F1-score (0.77 and 0.78, respectively) by WE. Similarly, NG again achieved the highest performance measures in identifying LOCATION. Although PS and WE had the same precision values as NG, their recall values and F1-score were less. A glance at the table confirms the consistent performance of NG across most of the PHI items.

**Table 1:** Precision, Recall, and F1-score achieved by the models trained by individual feature for each PHI.

**PHI**	**Performance measure**	**BC**	**NG**	**PS**	**RI**	**S**	**SP**	**DWE**	**WE**	**WS**	**WRE**
NAME	P	0.57	**0.80**	**0.79**	0.40	N/A	N/A	0.58	0.78	**0.79**	0.34
	R	0.36	**0.78**	0.75	0.21	0	0	0.44	**0.77**	0.75	0.06
	F1	0.44	**0.79**	0.77	0.28	0	0	0.50	**0.78**	0.77	0.11
LOCATION	P	0.78	**0.85**	**0.85**	0.81	N/A	N/A	**0.84**	**0.85**	**0.84**	0.10
	R	0.72	**0.82**	**0.81**	0.75	0	0	0.75	**0.81**	0.80	0.01
	F1	0.75	**0.84**	**0.83**	0.78	0	0	0.79	**0.83**	0.82	0.01
DATE	P	0.74	**0.85**	**0.85**	0.72	N/A	N/A	0.76	**0.84**	**0.85**	0.80
	R	0.45	**0.78**	**0.78**	0.45	0	0	0.45	**0.77**	**0.78**	0.70
	F1	0.56	**0.82**	**0.81**	0.55	0	0	0.56	0.80	**0.82**	0.74
HOSPITAL	P	0.82	**0.95**	**0.95**	0.78	N/A	N/A	**0.90**	**0.95**	**0.95**	0.05
	R	0.77	**0.93**	**0.92**	0.74	0	0	0.85	**0.93**	**0.92**	0.01
	F1	0.79	**0.94**	**0.93**	0.76	0	0	0.88	**0.94**	**0.93**	0.01
PHONE	P	N/A	**0.95**	**0.95**	**0.94**	N/A	N/A	0.93	**0.94**	**0.95**	**0.94**
	R	0	**0.94**	**0.94**	**0.94**	0	0	**0.94**	**0.94**	**0.94**	**0.94**
	F1	0	**0.94**	**0.94**	**0.94**	0	0	**0.94**	**0.94**	**0.94**	**0.94**
ID	P	0.98	**0.99**	0.97	N/A	N/A	N/A	0.64	0.92	**0.98**	0.96
	R	0.02	0.78	**0.80**	0	0	0	0.03	0.78	**0.81**	0.29
	F1	0.04	0.87	**0.88**	0	0	0	0.06	0.84	**0.89**	0.44
INITIALS	P	0.91	0.96	**0.97**	**0.98**	N/A	N/A	0.91	0.90	**0.97**	0.70
	R	0.08	**0.48**	0.45	0.06	0	0	0.12	0.19	**0.46**	0.10
	F1	0.14	**0.64**	**0.62**	0.13	0	0	0.22	0.32	**0.62**	0.02

*The highest and the second highest values of each performance measure for each PHI are marked by red and green colors, respectively*.

**Table 2:** Precision, Recall, and F1-score achieved by the models trained by all the features and four of the best performing features.

**PHI**	**Performance measure**	**All features combined**	**NG, PS, WE, and WS combined**
NAME	P	0.81	0.80
	R	0.80	0.79
	F1	0.81	0.80
LOCATION	P	0.87	0.85
	R	0.84	0.83
	F1	0.85	0.84
DATE	P	0.86	0.86
	R	0.78	0.79
	F1	0.82	0.82
HOSPITAL	P	0.95	0.96
	R	0.94	0.93
	F1	0.95	0.95
PHONE	P	0.97	0.95
	R	0.96	0.94
	F1	0.96	0.95
ID	P	0.98	0.99
	R	0.82	0.82
	F1	0.89	0.90
INITIALS	P	0.98	0.97
	R	0.47	0.49
	F1	0.64	0.65

However, NG did not yield the highest performance measures in some cases. For example, the values of recall and F1-score in ID were much less for NG than others. WS had the highest recall and F1-score of 0.81 and 0.89, respectively, and PS took the second position with 0.80 and 0.88, respectively. Similarly, NG could not perform well to predict INITIALS, as shown by its low precision. The above results showed that no single feature can be relied on to build a de-identification NER model. By analyzing the results presented in Table 1, we concluded that the best performing features were NG, PS, WE, and WS.

Table 2 compares the performance of the models trained by the combination of all the features and four best-performing features. The table shows no significant difference in the values of performance measures of these two models. In most cases, the combination of all features yielded a slightly higher value than the combination of the best four. For example, precision, recall, and F1-score of all feature combinations were 0.81, 0.80, and 0.81 for NAME, respectively, whereas the best four combinations had values of 0.80, 0.79, and 0.80, respectively. On the other hand, the best four combinations yielded higher precision, F1-score (0.99 and 0.98), than those of all feature combinations (0.98 and 0.89) for ID. Similarly, the best four combinations had higher recall and F1-score (0.49, 0.65) than all feature combinations (0.47 and 0.64) for INITIALS.

**Figure 3:**
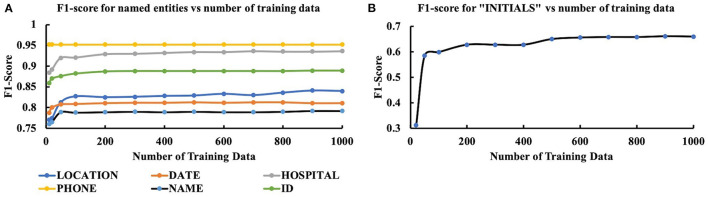
Variation of performance (F1-score) of CRF model with the size of training data for **(A)** LOCATION, DATE, HOSPITAL, PHONE, NAME, ID, and **(B)** INITIALS.

The results from the second stage of the experiment in which we investigated the variation of model performance with training set size are presented in Figure 3. Instead of considering all the performance measures, we evaluated the model's performance based on the F1-score. As Figure 3 suggests, F1-score was initially low but increased as the number of training data increased and eventually reached a saturation level for all the PHI items. For all PHI items, except LOCATION and INITIALS, F1-score got saturated after reaching a training sample size of 200. For LOCATION, initially, a saturation level was achieved after reaching a training sample size of 200, but the performance deteriorated for 700 training data and again came back to saturation level after 800. Although the exact reason for such aberrant behavior is unknown, a possible explanation could be the encounter of a complex training batch where the LOCATION context was somewhat different from other training batches. For INITIALS, the performance of our model was not satisfactory since the highest F1 value achieved was 0.64. While for all other PHI items, the lowest F1-score was 0.74 (NAME) after the first round of training with 10 data, it was about 0.31 for INITIALS. Such performance discrepancies would require further investigation.

## Discussion

This study was designed in two stages. At the first stage, we evaluated the performance of the machine learning algorithm, CRF, by using multiple features and determining the features that performed the best in annotating PHI in pathology reports. CRF is an algorithm that has been used in most of the top ranked NER systems. Since the performance of a supervised machine learning model depends largely on the selection of features, the objective of this experiment was to find out the best features that facilitate model's performance in recognizing the named entities.

We found that all the models trained by both the individual features and a combination of the features had a low F1-score for INITIALS. This poor performance can be attributed to the nature of the entity. In the pathology reports we used, INITIALS appeared mostly in isolated positions with no left or right members, making it difficult to contextualize. It is an indication that supervised NER models cannot be reliable for such out-of-context entities and would require further research on feature extraction. Although, it performed poorly in annotating INITIALS, this model outperformed few reported models in annotating other entities. A study that used MIST and an in-house system with pre-processing and post-processing steps reported an F1-score of 0.77 for LOCATION and 0.92 for PHONE NUMBER (27). Another study that compared the performance of three different tools: Amazon Comprehend Medical PHI, Clinacuity's CliniDeID, and the National Library of Medicine's Scrubber on two of the publicly available corpora: 2014 and 2016 i2b2 de-identification challenge corpora reported a maximum F1-score of 0.92 (2014), but a lower score of 0.88 on 2016 corpora in annotating ID (28).

The pathology reports in this study had various identifiers, but all the identifiers were not the PHI item, ID. Similarly, there were multiple abbreviations which were not INITIALS. Many of these identifiers and abbreviations were used to identify various specimens. Table 2 shows that for these two PHI items, the precision value was significantly higher than the recall. While annotating ID, the best four features yielded a precision of 0.99, whereas the recall value was 0.82. Which means that our model correctly identified 82% of all the IDs and 99% of the items that were identified as ID were actually the IDs. Similarly, a precision of 0.97 and a recall of 0.49 mean that even though only 49% of the INITIALS were correctly identified, 97% of the items identified as INITIALS were truly the INITIALS. A closer inspection of Table 2 reveals that for all of the PHI items, our model yielded a higher precision value than the recall. Such consistent higher precision values indicate that the model using the best four features may not be able to identify all the true PHI items, but majority of the identified items were correct.

At the second stage of the experiment, our objective was to determine the dependency of the model (using the best four features) on the size of the training dataset. The result of this analysis showed that the performance of the model reached a saturation point after 200 training data. After this point, no significant variation was observed in the model output with increased training data size. In this analysis, one interesting thing was that even for very few training data (*n* = 20), the model yielded a minimum F1-score of 0.74 (NAME) for all the entities except INITIALS. The model produced an F1-score of 0.95 for PHONE NUMBERS with only 20 training data. Such high F1 values for small training data size suggest the efficacy of appropriate features.

Results presented in Table 2 show no significant difference among the values of performance measures yielded by all-features-model and best-four-features-model. Table 1 shows that S and SP failed to annotate any PHI items and overall, all the features were outperformed by the best four features. Therefore, the high values of the performance measures of the all-features-model can exclusively be attributed to the best four features. Moreover, the use of the combination of all features made the training process more time-consuming. Hence, based on our analysis, it can be concluded that NG, PS, WE, and WS are the best features in building a clinical de-identification NER model.

**Figure 4:**
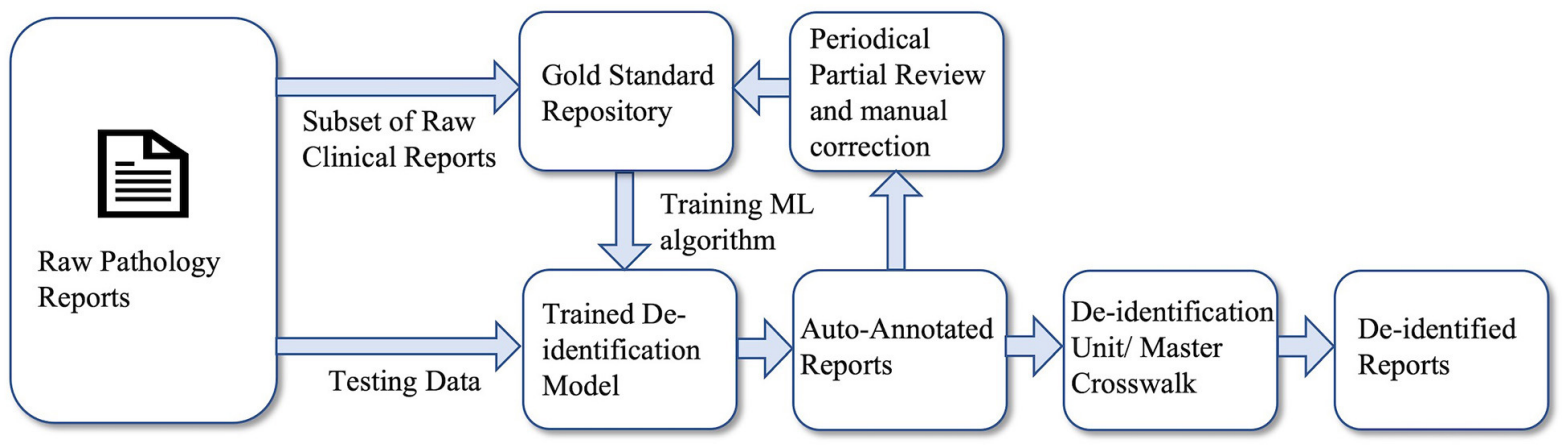
Framework for automatic clinical de-identification pipeline.

The analysis done in this study provides us with the significant knowledge required to maintain a data warehouse. Here, we present a framework for a clinical de-identification pipeline for enterprise-wide data warehousing which is illustrated in Figure 4. A subset of the raw clinical reports will be manually annotated, which will serve as the gold standard repository, a reliable, and accurate reference of the warehouse. These annotated data will be used in training the ML algorithm to build a NER model, which is the core unit of this pipeline. The model adds the automation feature to this pipeline as it will annotate the raw text reports without manual labor. The task of the de-identification unit is to replace the annotated entities either with information describing the type of entities ([NAME], [ADDRESS], [DATE], and [IDENTIFIER], etc.) or pseudonyms by applying master crosswalk. For enterprise-wide data warehousing for clinical text, the quality of the de-identification pipeline, in terms of prediction accuracy, has to be monitored periodically, and performance drift needs to be calibrated as well. The performance of the model will periodically be evaluated by reviewing a subset of the auto-annotated clinical reports. These reports will be manually corrected and added to the repository for performance calibration. Periodical performance evaluation and correction ensure the reliability of this automated clinical de-identification pipeline.

A limitation of this study is that it was conducted using only one type of clinical narrative from a single source. The organization of the reports may significantly vary depending on the type and source of the reports, which may change the values of the performance measures. Moreover, some of the high values of the performance measures, in this study, may be attributed to the homogeneity of the data. A future study on more heterogeneous corpora from multiple sources may confirm whether or how the performance of the models will vary.

## Conclusion

In this study, we built CRF-based NER models for clinical de-identification by using multiple features and their combinations. Our objective was to evaluate the performance of these models and find out the best performing features. By exploiting the feature extracting function provided by the CLAMP toolkit, we concluded that the best-performing feature extractors are n-gram, prefix–suffix, word embedding, and word shape. We did further analysis to observe the dependency of the model on the number of training data, and we noticed that a saturation level is reached beyond 200 training data. Based on the results of these analyses, we presented a framework for the clinical-deidentification pipeline, which facilitates enterprise-wide data warehousing.

## Data Availability Statement

The data analyzed in this study is subject to the following licenses/restrictions: HIPAA Compliance. Requests to access these datasets should be directed to mosaa@health.missouri.edu.

## Author Contributions

AM was the principal investigator of this project. It was conceptualized and designed by AM, RH, VS, and MB. Data pre-processing and technology setup for data annotation was conducted by MR and VM. PT, TK, YJ, and NS did the data annotation. TP built the machine learning models, analyzed their performances, and prepared the first draft of the manuscript. Project management was done by AM and VM. All authors provided their critical reviews and contributed to writing.

## Funding

This study was funded by Roche Diagnostics Information Solutions. The funder had the following involvement with the study: study design and manuscript review.

## Conflict of Interest

VS and MB were employed by F. Hoffmann-La Roche. The remaining authors declare that the research was conducted in the absence of any commercial or financial relationships that could be construed as a potential conflict of interest.

## Publisher's Note

All claims expressed in this article are solely those of the authors and do not necessarily represent those of their affiliated organizations, or those of the publisher, the editors and the reviewers. Any product that may be evaluated in this article, or claim that may be made by its manufacturer, is not guaranteed or endorsed by the publisher.
